# Comparison of Systemic and Mucosal Synchronous Immunization with Replicating Single-cycle Adenoviruses and SOSIP Protein HIV-1 Vaccines

**DOI:** 10.21203/rs.3.rs-10702892/v1

**Published:** 2026-08-26

**Authors:** Pramod N Nehete, Bharti P Nehete, Kathryn Shelton, Haley E. Mudrick, Mary E. Barry, Guojun Yang, Stephanie Dorta-Estremera, Shilpi Pandey, Peng Xiao, Shaunna Shen, David C. Montefiori, Francois Villinger, Ann J. Hessell, Nancy L. Haigwood, K. Jagannadha Sastry, Michael A. Barry

**Affiliations:** 1 Department of Comparative Medicine for The University of Texas M.D. Anderson Cancer Center, Houston and Bastrop, TX; 2 Department of Michael Keeling Center for Comparative Medicine and Research for The University of Texas M.D. Anderson, Cancer Center, Houston and Bastrop, TX; 3 Molecular Pharmacology and Experimental Therapeutics Graduate Program, Mayo Clinic, Rochester, MN; 4 Department of ORBIT, and Immunology for The University of Texas M.D. Anderson Cancer Center, Houston and Bastrop, TX; Department of Internal Medicine, Division of Public Health Occupational Medicine and Infectious Diseases, Mayo Clinic, Rochester, MN; 5 Department of UTHealth Graduate School of Biomedical Sciences at Houston for The University of Texas M.D. Anderson Cancer Center, Houston and Bastrop, TX; Oregon Health & Sciences University, Portland OR; 6 Division of Pathobiology and Immunology, Oregon National Primate Research Center; 7 Department of Biology, New Iberia Research Center, University of Louisiana at Lafayette, New Iberia, LA.; 8 Laboratory for HIV and COVID-19 Vaccine Research and Development, Department of Surgery, Duke University Medical Center Durham, North Carolina; 9 Department of Immunology, Department of Molecular Medicine, Mayo Clinic, Rochester, MN

## Abstract

Most HIV-1 infections occur at mucosal surfaces. A small number or single incoming virions start each infection and then these amplify exponentially, spread through mucosal tissues and then throughout the body. It is appealing to attempt to stop the infection during those first mucosal infection events when there are fewer viruses to combat. Most vaccines are injected intramuscularly (IM) to drive systemic and mucosal responses. However, data suggests that immunization at mucosal surfaces can build better mucosal barriers than systemic immunization. To explore this question, we performed synchronous immunizations of rhesus macaques with gene-based replicating single-cycle adenovirus (SC-Ad) expressing SIV gag and clade C gp160 envelope, and SOSIP clade C envelope protein vaccine. SOSIP vaccine was delivered by the IM route whereas SC-Ad was delivered by the systemic IM route or by mucosal intranasal (IN) or mucosal intravaginal (IVAG) injections. The same species C Ad6 serotype of SC-Ad was used for all four immunizations by shielding its surface with polyethylene glycol (PEG). 50% of IVAG immunized animals resisted vaginal clade C SHIV 1.5 years after last vaccination. In contrast, IN and IM vaccinated animals were infected with the virus within 10 weeks. These data suggest that vaccination at the mucosal portal of infection provides better localized protection against incoming SHIV and perhaps HIV-1 than standard intramuscular vaccination against repeated mucosal exposure to primate lentiviruses.

## Introduction

As much as 90% of HIV-1 infections occur by sexual transmission at mucosal surfaces (1) where only one or only a few virions infect the host (2), Experts on the initial events of HIV infection consider this a pivotal point to halt HIV infection (2), This supports the premise that an HIV vaccine that can neutralize these few virions or infected cells at the mucosal entry may be a potent feature for an HIV-1 vaccine, An ideal vaccine may generate barrier protection and robust systemic immune responses to control viruses that manage to bypass the barrier.

The unique biology of the mucosal immune system allows one to deliver vaccines to readily accessible nasal or oral sites to elicit responses at sites that are less addressable, but more relevant for protection against HIV (e.g. antibody and cellular immune responses in the vagina or rectum) (3), Systemic immunization by intramuscular (IM) injection can drive mucosal immune responses, This premise is supported by work by many groups including by Drs. Barouch and Ertl (4–7),

However, mucosal immunization may better educate immune cells to target and persist at mucosal surfaces (reviewed in (8)), For example, most mucosal cytotoxic T lymphocytes (CTLs) begin their development in local lymphoid tissues and move into mucosa during immunization, but only small numbers of circulating T cells enter mucosa and most are excluded from the epithelial layer and may be trapped in capillaries (8).

Among experimental vaccine attempts, adenovirus vaccines have been attempted owing to their ease of production, scalability and immunogenic properties (4, 5, 9–13). While initially promising, these approaches have delivered disappointing results due to a variety of factors (*i.e.* replication-defective Ad5 (Ad5) STEP trial lack of efficacy and increased acquisition; Ad26 Imbokodo trial with low efficacy; Ad26 Mosaico Trial with failure to reduce HIV acquisition (14, 15) (16–19)).

These published adenovirus vaccine trials utilize E1-deleted replication-defective Ads (RD-Ads) (4–7). An E1-deleted Ad vaccine infects a cell, delivers its one copy of an HIV-1 antigen gene, and expresses “1X” of this HIV protein, These vaccines are safe, but are not actually the most potent adenovirus vaccine platform, since they do not replicate their transgenes to amplify antigen expression. The most potent adenovirus vaccines are in fact E1-intact replication-competent adenoviruses (RC-Ads). These vaccines deliver the HIV genes to cells, but instead of only transcribing them, they also replicate the antigen gene DNA up to 10,000 times to produce substantially more antigen and induce markedly stronger immune responses than published E1-deleted RD-Ad HIV vaccines (9–11, 20–40).

While RC-Ad vaccines are documented to be more potent than RD-Ad vaccines, replication-competent Ads run the real risk of causing frank adenovirus infections in humans (41, 42).

Our laboratory developed single-cycle Ad (SC-Ad) vectors to harness the ability to replicate antigen genes like RC-Ad, but that are disabled from producing infectious progeny viruses to avoid the adenovirus infection side effect (43, 44), SC-Ads produce 100-fold higher protein amounts than benchmark RD-Ads (44–47) and generate more robust and persistent immune responses than RD-Ads (44, 45, 48). SC-Ads generate antibodies and T cells against HIV, influenza, Ebola, Zika, *C. difficile* antigens and SARS-CoV-2 antigens that persist for over 12 months after single immunization (46–51). SC-Ad mediated better protection vs *C. difficile* than *C. difficile* protein vaccine (48). SC-Ad expressing the SARS-CoV-2 Spike generated significantly higher antibodies than RD-Ad-Spike after single IN or IM and mediated better protection against SARS IN challenge more than 10 months after single immunization (47). When SC-Ad-Spike was compared to replication-competent vesiculostomatitis virus (VSV) pseudotyped with Spike, the highest IgA and CD8 T cells were observed in BAL when SC-Ad was used as a single vaccine or as a prime-boost combination with VSV (52).

SC-Ad6 and SC-Ad657 were used to express gp160 in rhesus macaques by IN or IM immunization (no gag, pol, or other SIV or HIV antigens were express) (50). HIV env plasma antibodies were significantly higher by IM than IN groups after single immunization. Each group was boosted by either the IM or the IN route to produce significant antibody and T cell responses. However, protein boosting with gp140 protein amplified antibody titers by 2 orders of magnitude. While binding and neutralizing antibodies were observed in plasma, there was an apparent “distance effect” in the levels of antibodies in the saliva and in vaginal samples depending on where the animals were vaccinated. Animals that received SC-Ad by the IM route had IgG at both mucosal sites. However, the animals immunized by the IN route only had Env-binding antibodies in their saliva near their mucosal nasal site of immunization, but markedly less in the distant vaginal mucosa. All of these animals became infected after rectal SHIV challenge. However, post-mortem sampling showed that animals immunized with SC-Ad by the mucosal intranasal route had lower levels of SHIV in their colons than other groups (53).

Given this mucosal distance effect observation, and the marked IM boosting effect of the protein vaccine, this study set out to compare IM and IN routes of SC-Ad vaccination with potentially “positive control” intravaginal (IVAG) immunization prior to a vaginal challenge with clade C SHIV. SC-Ad6 expressing clade C HIV gp160 with SC-Ad expressing SIV gag were delivered by these different routes and were combined in a co-immunization strategy (54, 55) with clade C stabilized SOSIP trimer (14–16). Macaques were immunized over several months. Notably, these animals were challenged vaginally with clade C SHIV more than 1.5 years after receiving their last immunization.

## Materials and Methods

### Single Cycle Adenoviruses.

SC-Ads retain their E1 gene to allow them to still replicate their DNA but have their pIIIA gene deleted to prevent them from producing infectious progeny viruses (43, 44). They are produced from 293-IIIA cells that stably express the Ad6 pIIIA gene with no genetic overlap between the cells and the viruses. Lower seroprevalence species C Ad6 was used rather than species C Ad5 vectors. Ad5 has 27% and 78% seroprevalence in Texas (56) and Belgium (57) whereas species C Ad6’s seroprevalence is 3% to 40% in the two sites. (54, 55). A codon-optimized clade C gp160 gene was used in SC-Ad. This gp160 amino acid sequence was identified as a transmitted/founder (T/F) envelope immunogen isolated as a week 54 early breadth from CAP257 subject in the CAPRISA study (58–60), Unlike clade B gp160, this clade C gp160 could not be rescued in standard SC-Ad, perhaps due to protein toxicity killing the producer cells. To circumvent this, the gene was inserted into a cassette downstream of the Rouse Sarcoma Virus (RSV) enhancer/promoter and a LoxP-Zeocin Resistance Gene-LoxP (RSV-LZL) cassette to inhibit expression during initial virus rescue in 293-IIIA cells. The virus was amplified up to 6 T225 flasks in 293-IIIA cells whose lysates were used to infect a 10 plate CellStack of 293-IIIA-Cre cells to delete the obstructing LoxP-ZeoR-LoxP cassette. A similar SC-Ad with RSV-LZL was constructed with SIV gag-XS (SIV-gag fused to 151 amino acids of RT) and was rescued similarly. Both viruses were purified from CsCl gradients, desalted, and their virus particle/ml (vp/ml) were quantified by OD_260_ as in (43, 44).

### SC-Ad PEGylation.

**Figure 1.**
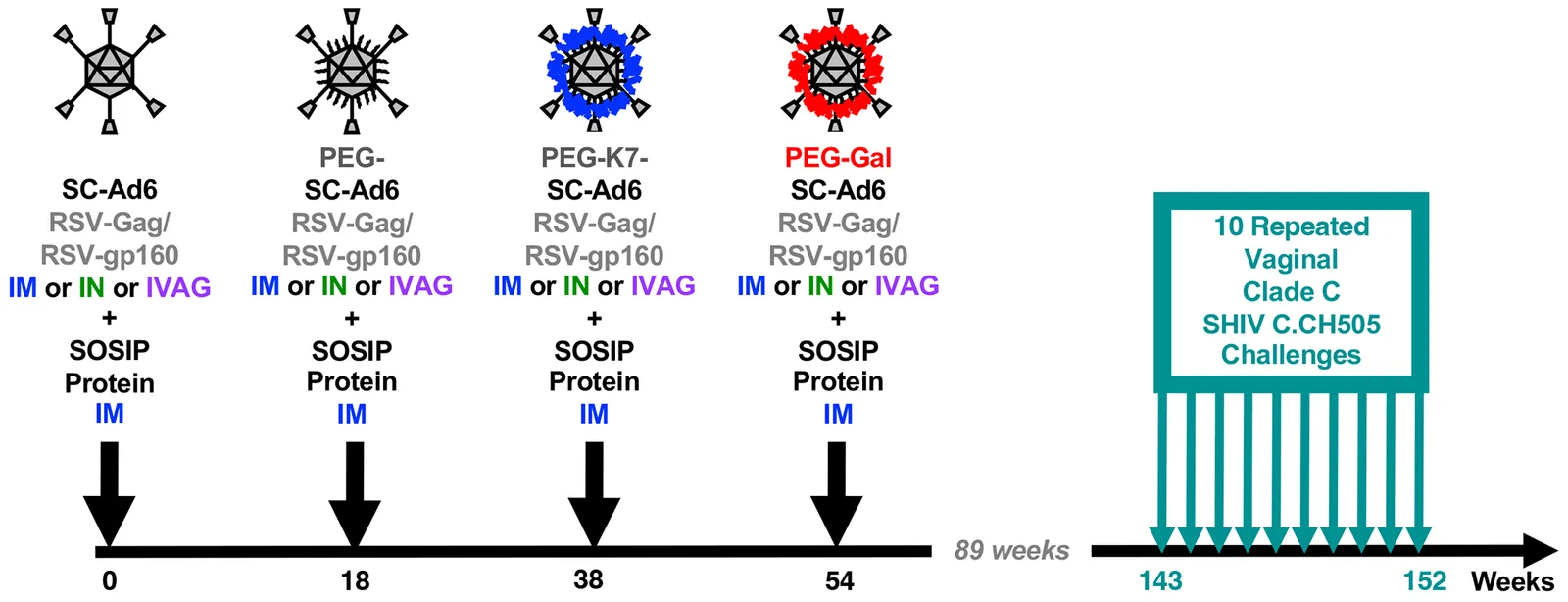
Vaccine Study Timeline and Interventions.

To reuse the same SC-Ad serotypes for the second, third, and fourth immunizations, the viruses were “shielded” with different polyethylene glycol (PEG) polymers (Figure 1 and (61–74)). A different terminal modification was used at each round of immunization to reduce the likelihood of neutralization by any PEG antibodies that might be produced. After their first CsCl spin, the viral bands were isolated and reacted with the different PEG molecules for 1 hour and then were spun a second time prior to desalting. For immunization number two, the SC-Ads were conjugated with monofunctional 5 kDa NHS-PEG as in (71, 75). For immunization three, the SC-Ads were reacted with heterobifunctional 5 kDa NHS-PEG-maleimide and then with the cysteine on a polylysine peptide (CKKKKKKK). For immunization four, the SC-Ads were reacted with 35 kDa PEG capped with galactose as in (75).

### Envelope Proteins.

Trimeric “native-like” clade C SOSIP protein was generously provided by Al Cupo and Dr. John Moore supported by NIH/NIAID P01 AI110657 (45–47). This clade C SOSIP was purified from CHO cells that expresses CZA97 SOSIP.v4.2-M6.IT (76).

### Animals.

Groups of 8 Adult female rhesus macaques (*Macaca mulatta*) of Indian origin were used for these studies at the Michael Keeling Center for Comparative Medicine and Research at the University of Texas MD Anderson Cancer Center, Bastrop, TX. All animal studies were approved and performed in accordance with the provisions of the Animal Welfare Act, PHS Animal Welfare Policy, the principles of the NIH Guide for the Care and Use of Laboratory Animals. ACUF protocol number?

### Co-Immunizations.

Macaques were anesthetized with ketamine and immunized intranasally (IN) with liquid vaccine or were injected intramuscularly (IM) in their deltoid muscle or were injected in their vaginal epithelium (IVAG) with 10^11^ virus particles (vp) each of SC-Ad-Gag and SC-Ad-Env vaccines. At the same time, the animals were immunized only by the IM route in the other deltoid muscle with 50 μg of SOSIP protein combined with Adjuplex adjuvant (77, 78). SOSIP was only delivered by the IM route since at the time, this was the only route that these immunogens had been tested.

### Sample Collections.

Peripheral blood mononuclear cells (PBMC) and plasma was separated from peripheral blood in EDTA and stored immediately at −80°C. PBMCs were prepared on Ficoll-Hypaque gradients. Saliva and vaginal swabs were collected with Wek-Cel Spears in 1 ml of PBS containing protease inhibitors, vortexed, and supernatants were collected after 2,000 rpm centrifugation as in (79). Samples were kept frozen at −80°C.

### ELISPOT Assay for Detecting Antigen-specific IFN-γ producing Cells.

Fresh PBMCs were stimulated with either F8 gp140 protein (1 μg/mL) or heat inactivated Ad6 (7.0 X10^8^ vp/well) to determine the numbers of IFN-γ-producing cells by the Enzyme Linked Immuno Spot (ELISPOT) assay using the methodology reported earlier (80–82). Briefly, aliquots of PBMCs (10^5^/well) were seeded in duplicate wells of 96-well plates (polyvinylidene difluoride backed plates, MAIP S 45, Millipore, Bedford, MA) pre-coated with the primary IFN-γ antibody and the lymphocytes were stimulated with either Con A, F8 gp140 protein, or heat inactivated Ad6. After incubation for 30-36 hours. at 37°C, the cells were removed and the wells were thoroughly washed with PBS and developed as per protocol provided by the manufacturer. Results are expressed as IFN- γ spot-forming cells (SFCs) per 10^5^ PBMCs after subtraction of the duplicate wells with medium only (negative control) and are considered positive if greater than twice the background and greater than 5 SFCs/10^5^ PBMCs.

### Antibody ELISAs.

HIV-1 envelope binding antibody midpoint binding titers were measured in plasma samples collected at regular intervals against clade C gp140 as described previously (77, 78). What about mucosal samples? Extraction method? test?

### Neutralization assay.

HIV neutralization was performed using the TZM-bl neutralization assay by Laboratory for HIV and COVID-19 Vaccine Research and Development at Duke as in (77, 78) under the supervision of Dr. Shaunna Shen and Dr. David Montefiori. All values were calculated as compared to virus-only wells.

### Antibody Dependent Cellular Cytotoxicity (ADCC).

CEM.NKR.CCR5.CD4+−Luc, target cells were infected with 50 ng SHIV_SF162P3_ and cultured for 4 days as in (83). Two-fold serial dilutions of each sample were added to the infected targets for 20 min at room temperature. CD16-KHYG-1 effector cells were added at a 10:1 effector to target ratio and these were incubated for additional 8 hours. The cells were lysed and luciferase activity was measured on the Bio-Tek plate reader.

### Flow Cytometry.

Cells collected from rectal and lymph node biopsies were incubated overnight with 0.2 μg gp140 or media alone in the presence of Golgi Plug for the last 4 hrs. After culture, cells were harvested and incubated on ice for 45 min with a panel of human antibodies that cross-react with rhesus macaque samples. The panels included the following fluorochrome labeled antibodies: CD8 (Qdot655), α4β7 (PE) and CXCR5 (PE), all obtained from the Nonhuman Primate Reagent Resource; CD69 (BV737, clone FN50) and FoxP3 (PECy5, clone: PCH101) obtained from ebioscience, IL-21 (BV421, clone: 3A3-N2.1), CD45 (BV786, D058-1283) and CD3 (clone SP34-2, PE-Cy7-labeled) all from BD Bioscience (San Jose, CA); CD4 (Pacific Blue, clone OKT4) from ThermoFisher Scientific (Waltham, MA). Dilutions for antibodies were determined by following manufacturer’s recommendations. Dead cells were excluded by using live-dead fixable dead cells stain kit obtained from Invitrogen (Carlsbad, CA), Subsequently, the cells were washed twice with PBS containing 2% FBS and 2 mM EDTA and then fixed and permeabilized with FoxP3 Fix/Perm Kit (ThermoFisher Scientific, Waltham, MA), The intracellular markers FoxP3 and IL-21 were stained in permeabilization buffer, Both compensation controls (OneComp eBeads, (ThermoFisher Scientific, Waltham, MA) and fluorescence minus one (FMO) controls were utilized, All the samples were collected on an LSR Fortessa X-20 analyzer (BD Biosciences, San Jose, CA) and were analyzed using FlowJo software (FlowJo, LLC, Ashland, Oregon), Approximately 2 x 10^5^ to 1 x 10^6^ events were collected per sample.

### Vaginal Challenge with Rhesus Macaque CD4-Binding Clade C SHIV.

Rhesus macaque CD4 binding C.CH505 SHIV virus developed by Dr. Shaw (84) was used for challenges. C.CH505 SHIV was generously provided Dr. Nancy Miller, NIH/NIAID. This stock had a P27 content of 66 ng/ml, RNA content Log ~9.35, TCID_50_ in Indian origin rhesus PBMC: 1288/ml, and TCID_50_ in TZM-bl cells: 4.1 x 10^4^/ml. 1 ml of a 1:300 dilution of the stock was used as recommended by Dr. Miller. This equaled 4.3 TCID_50_ on rhesus PBMCs and 137 TCID_50_ on TZM-bl cells. This dose was used for weekly intravaginal (IVAG) challenge, Animals with plasma RNA copies above 10 were considered to be infected and the number of challenges required to infect that animal were used as events for Kaplan-Meier survival analysis. Once infected, the animal was no longer challenged. Plasma viral loads were monitored weekly and later monthly by the same method until the end of the study.

### Plasma SHIV Viral Loads.

Plasma samples were analyzed for SHIV viral RNA copy numbers by Leidos Biomedical Research, Inc., Frederick National Laboratory.

### Statistical Analysis.

GraphPad Prism software was used for statistical analyses,

## Results

### Synchronous SC-Ad and SOSIP Vaccination against Clade C SHIV by Different Routes of Immunization.

We utilized a co-immunization strategy that had shown some effectiveness in primates using a different adenovirus vector (85). Female rhesus macaques were immunized with 10^11^ vp of SC-Ads expressing SIV gag and clade C HIV-1 gp160 Env (Fig. 1). SC-Ads were delivered by different routes including intramuscular (IM) injection into the deltoid muscle, intranasal (IN), and intravaginal (IVAG) routes. Our prior work showed that liquid immunization in the vaginal tract was ineffective (86), so IVAG group animals had SC-Ads injected directly into the vaginal epithelium at the portal of SHIV entry as in (86). The animals were also concurrently immunized with 50 μg of clade C SOSIP CZAv4.2-M6.IT protein trimers adjuvanted with Adjuplex, but only by the IM route, since this was the only route previously used for this protein vaccine. The SC-Ad clade C gp160 and the clade C SOSIP protein had 81.4% amino acid identity (Supplemental Figure 1).

The animals were immunized four times with the same human adenovirus serotype 6 (Ad6) SC-Ad. Ad6 and Ad5 are both robust species C human adenovirus vaccines, but Ad6 has only about 50% of the Ad5 seroprevalence in humans (56, 57, 87). To avoid pre-existing immunity to SC-Ad6 serotypes, we “shielded” the viruses by covalently modifying their surfaces with different polyethylene glycol (PEG) polymers (61)(Fig. 1). SC-Ads were PEGylated with NHS-PEG that crosslinks lysines on the viral surface. For the second immunization, 5 kDa PEG alone was used for shielding. For the third immunization. PEG-K7 with 7 lysines on its end was conjugated to change the PEG surface and to bind to cellular heparin sulfate proteoglycans. For the last SC-Ad immunization, the vaccine was conjugated to PEG-Gal with a galactose on its end to carbohydrate receptors. This PEG modification was previously shown to improve IN immunization by Ad5 vaccines (74).

### Immune Responses after SC-Ad and SOSIP Co-immunization Prime and Boost.

**Figure 2.**
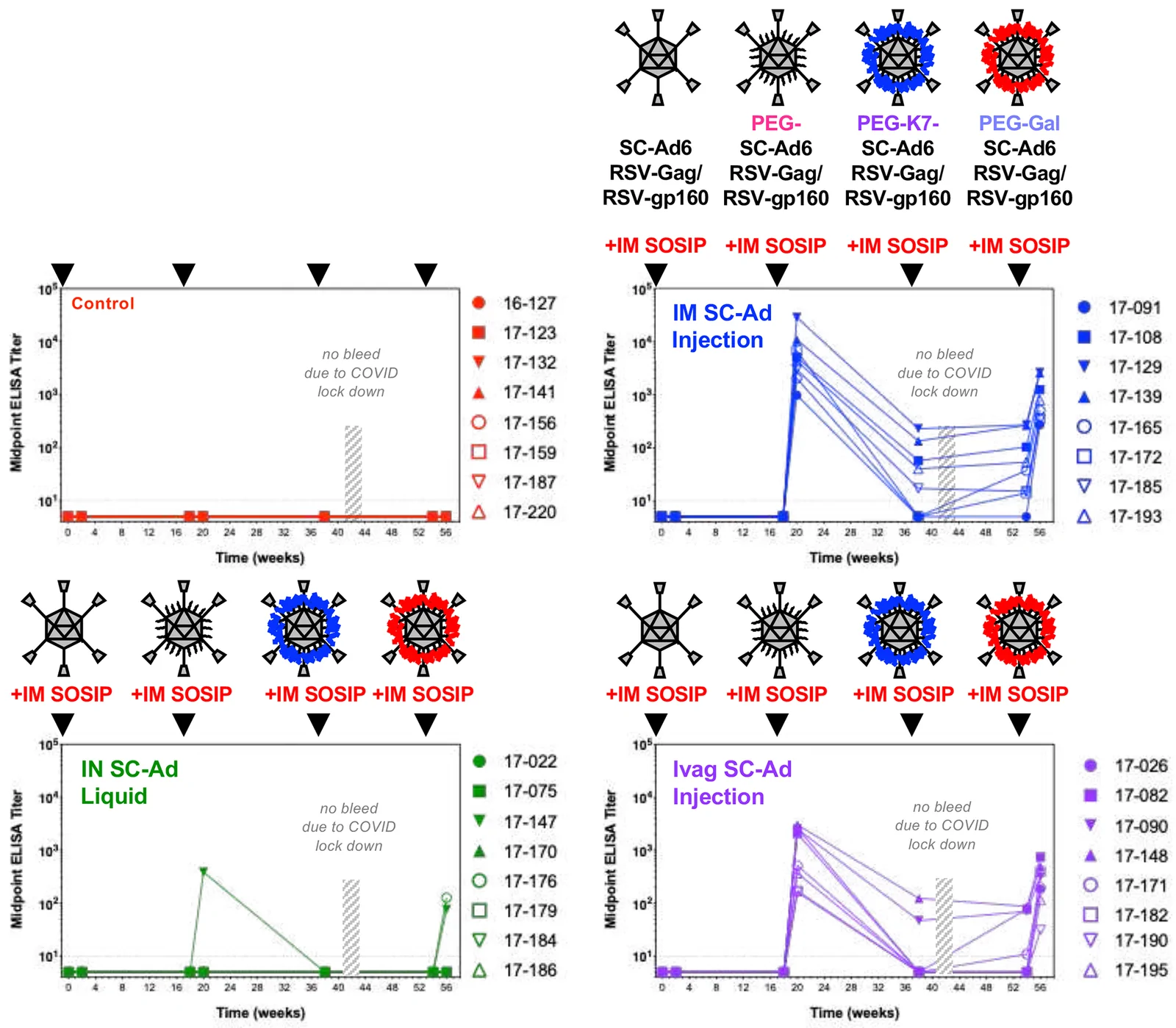
Plasma HIV Env Binding Titers. Immunizations with different SC-Ads and gp140 proteins are shown above the graph with large arrows. Midpoint env binding titers by ELISA are shown for each animal before and after each immunization.

The single co-immunization of SC-Ad6-gag, SC-Ad6-gp160 and clade C SOSIP protein generated minimal env antibodies in plasma irrespective of the vaccination route (Fig. 2). After the booster with PEG-SC-Ad and SOSIP 15 weeks later, antibody titers climbed 10 to 1000-fold in the IM and IVAG SC-Ad injected animals (Fig. 2). Surprisingly, only one animal in the IN SC-Ad + IM SOSIP group generated antibodies after two immunizations.

**Figure 3.**
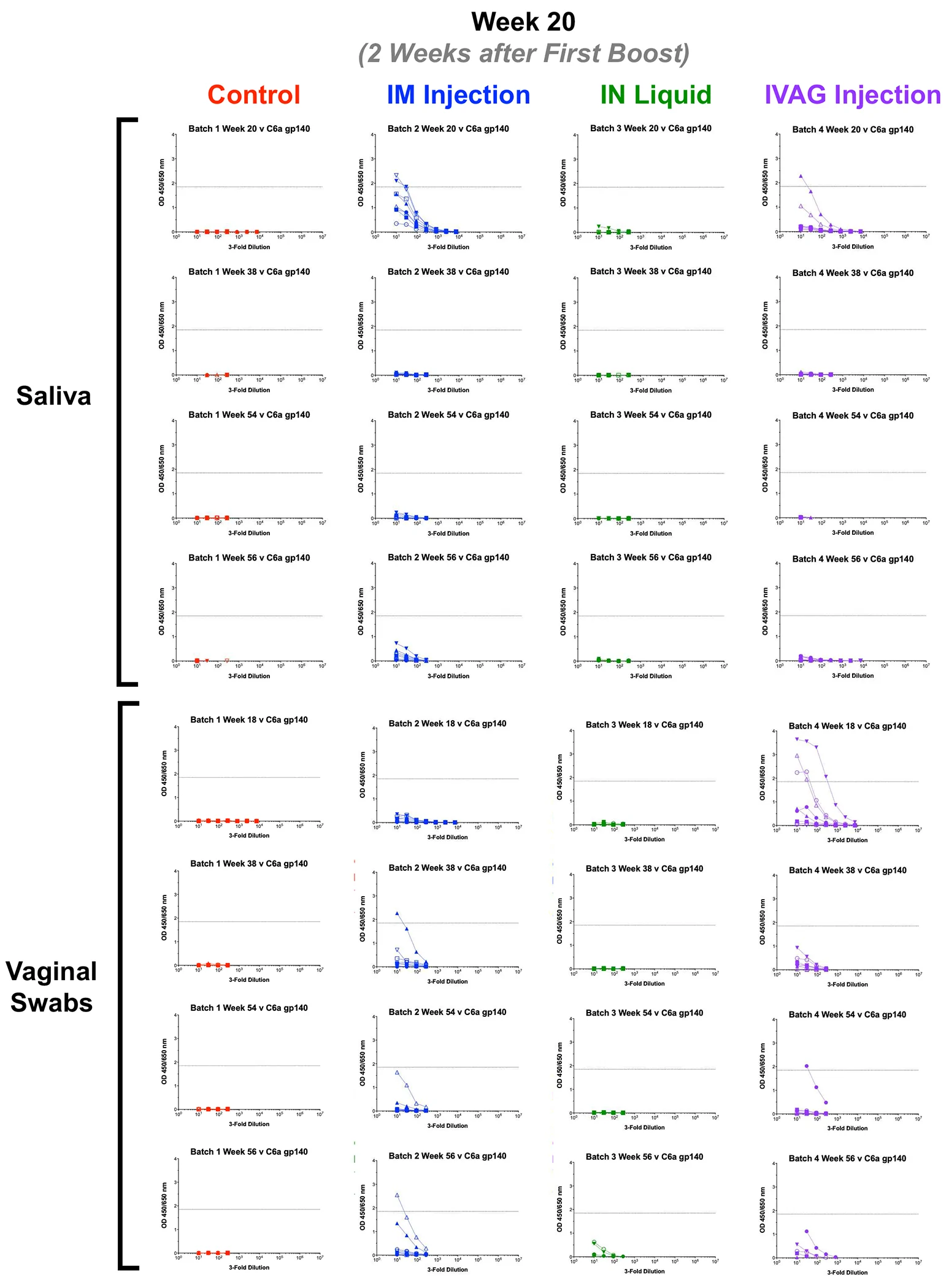
Salivary and Vaginal Sample HIV Envelope ELISA Binding after Two Immunizations. Env-binding ELISA optical density measurements are shown as lines for each individual animal’s saliva and vaginal samples. Lines represent different optical densities at different dilutions of each sample.

These antibodies were measured in plasma which may not be relevant to protection at the mucosal barriers where HIV and SHIV enter the body. To explore mucosal immune responses, salivary and vaginal swabs were tested for antibodies. After two immunizations, all IM and 2 out of 6 IVAG SC-Ad injected animals showed detectable env binding antibodies in saliva samples (Fig. 3) though fewer animals exhibited detectable vaginal antibodies from the same groups. In contrast, animals vaccinated with IN SC-Ad plus IM SOSIP showed no detectable mucosal antibodies at any time (Fig. 3).

### Antibody Responses after Four SC-Ad and SOSIP Co-immunizations

**Figure 4.**
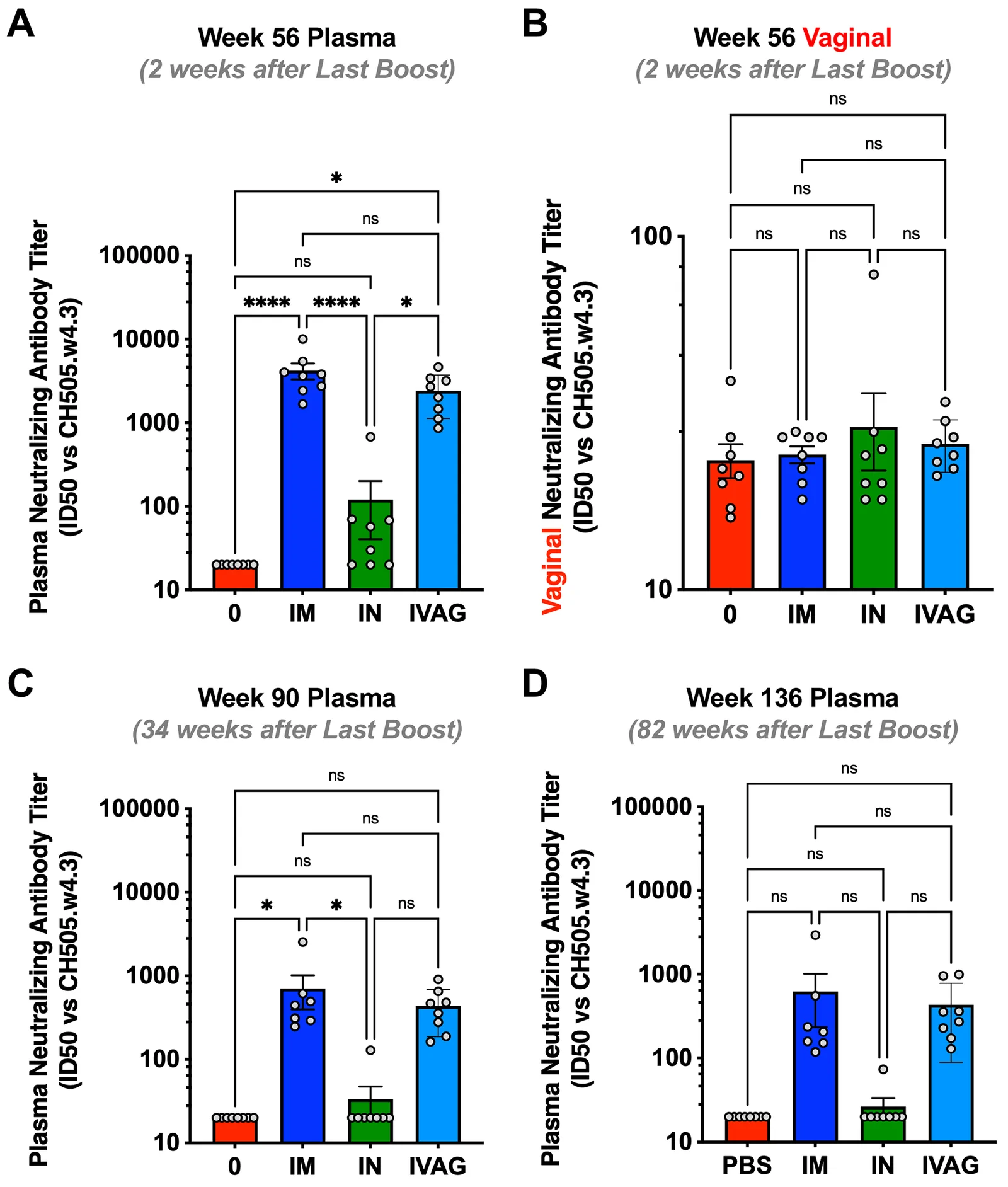
Plasma Clade C SHIV Neutralizing Antibodies. Neutralization of clade C SHIV CH505.w4.3 was performed using the TZM-bl neutralization assay. All values were calculated as compared to virus-only wells. Each dot represents the mean value for each animal. * p < 0.05 and **** p < 0.0001 by one-way ANOVA.

**Figure 5.**
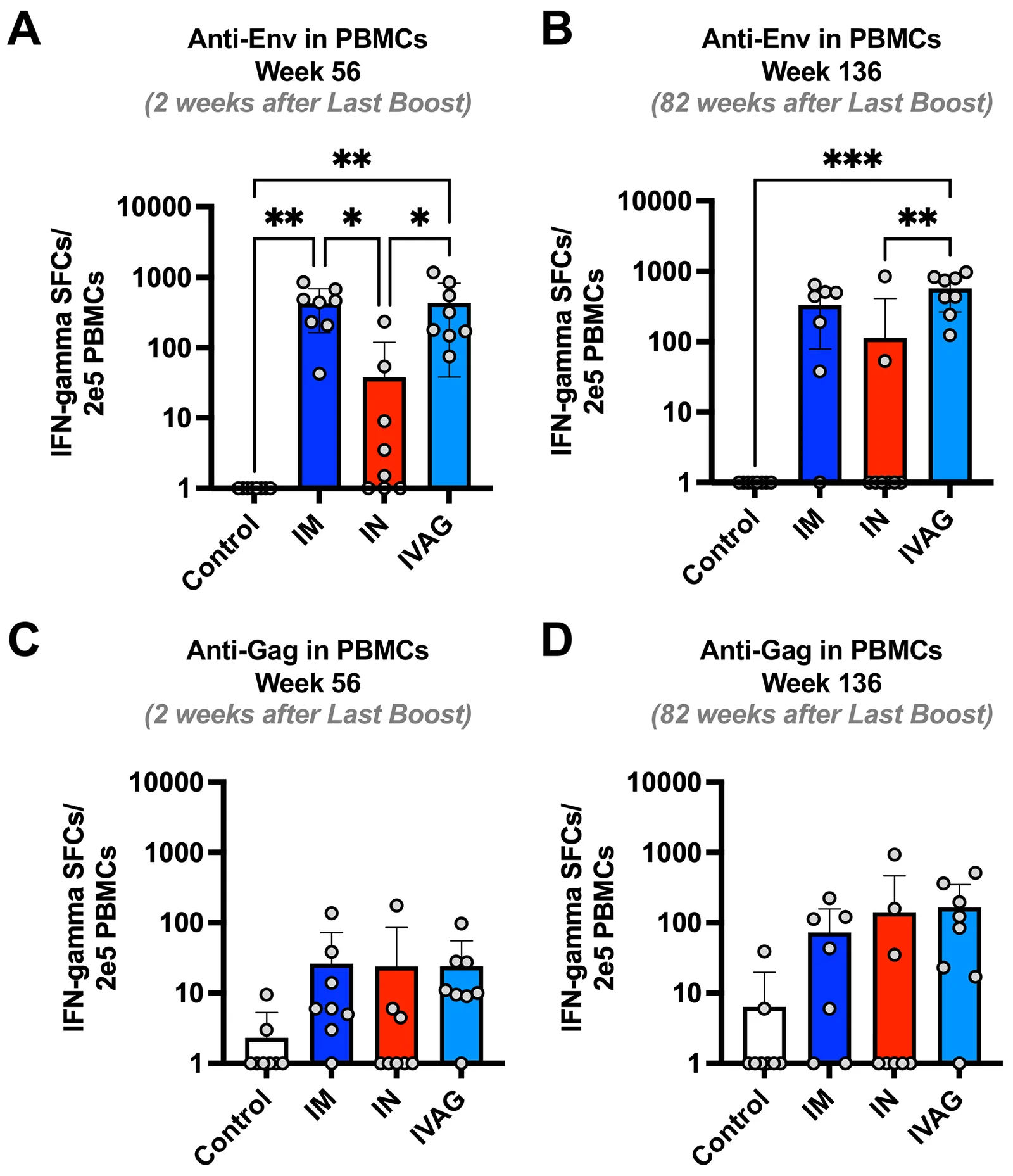
IFN-γ Secreting Cells in PBMCs. PBMCs were analyzed by ELISPOT by staining for IFN-γ. Anti-Env indicates cells that were stimulated with a conserved clade C HIV Env peptide pool. Anti-gag indicates cells stimulated with a SIV gag peptide pool. The total number of spot forming cells (SFCs) in each of the stimulated wells were counted and adjusted to control medium as background. Each dot represents the mean value for each animal. * p < 0.05, ** p < 0.01, *** p < 0.001 by one-way ANOVA.

The animals were subsequently co-immunized 38 and 54 weeks after first immunization (Fig. 1). Animals received SC-Ads conjugated with PEG-K7 at week 38 and conjugated with PEG-Gal at week 54 by the same vaccine routes along with IM SOSIP co-immunizations. Samples were not collected immediately between weeks 38-54 due to facilities access restrictions during the COVID-19 pandemic. At week 54, 16 weeks after the last boost, approximately half of IVAG and most of the IM SC-Ad animals still had env binding antibodies in their plasma (Fig. 2) which were boosted by the last immunization at week 54. In contrast, IN SC-Ad plus IM SOSIP animals did not have significant plasma env antibodies by ELISA after 3 and even 4 immunizations. Week 56 plasma samples were tested for clade C SHIV NAbs, revealing significant levels of Tier 1 (but not Tier 2) clade C SHIV neutralizing antibodies in IM and IVAG-immunized animals (Fig. 4A). These titers while decreasing from the wk 56 peak responses showed remarkable longevity over the subsequent 1.5 year (Fig. 5C, D). In correlation with binding antibody results, the IN group failed to produce even tier 1 directed neutralizing antibodies. Week 56 vaginal samples also failed to exhibit SHIV NAbs despite detectable binding antibodies (Fig. 4B).

### T Cell Responses after Four SC-Ad and SOSIP Co-immunizations

Antigen specific T cells were tested by ELISPOT from week 56, and 136 PBMCs (2 and 82 weeks after the last boost, respectively) against clade C envelope and SIV gag peptides (Fig 5). Week 56 PBMCs showed significant IFN-y-spot forming cells (SFCs) reactions to clade C env for both the IM and IVAG SC-Ad groups (Fig. 5A and data not shown), which showed only minimal decrease 1.5 years later (Fig 5B). Anti-env SFC responses were weaker in the IN SC-Ad plus IM SOSIP group, though the difference was not significant. Of interest was the observation that these responses too were durable and even increased slightly over time (Fig 5B). SIV gag ELISPOT responses were surprisingly weaker than env responses though also durable and showing increases over time, likely owing to the particular SC-Ad vector used (Fig. 5C, D).

### Vaginal Immune Cells 82 weeks after Two SC-Ad and SOSIP Co-immunizations

**Figure 6.**
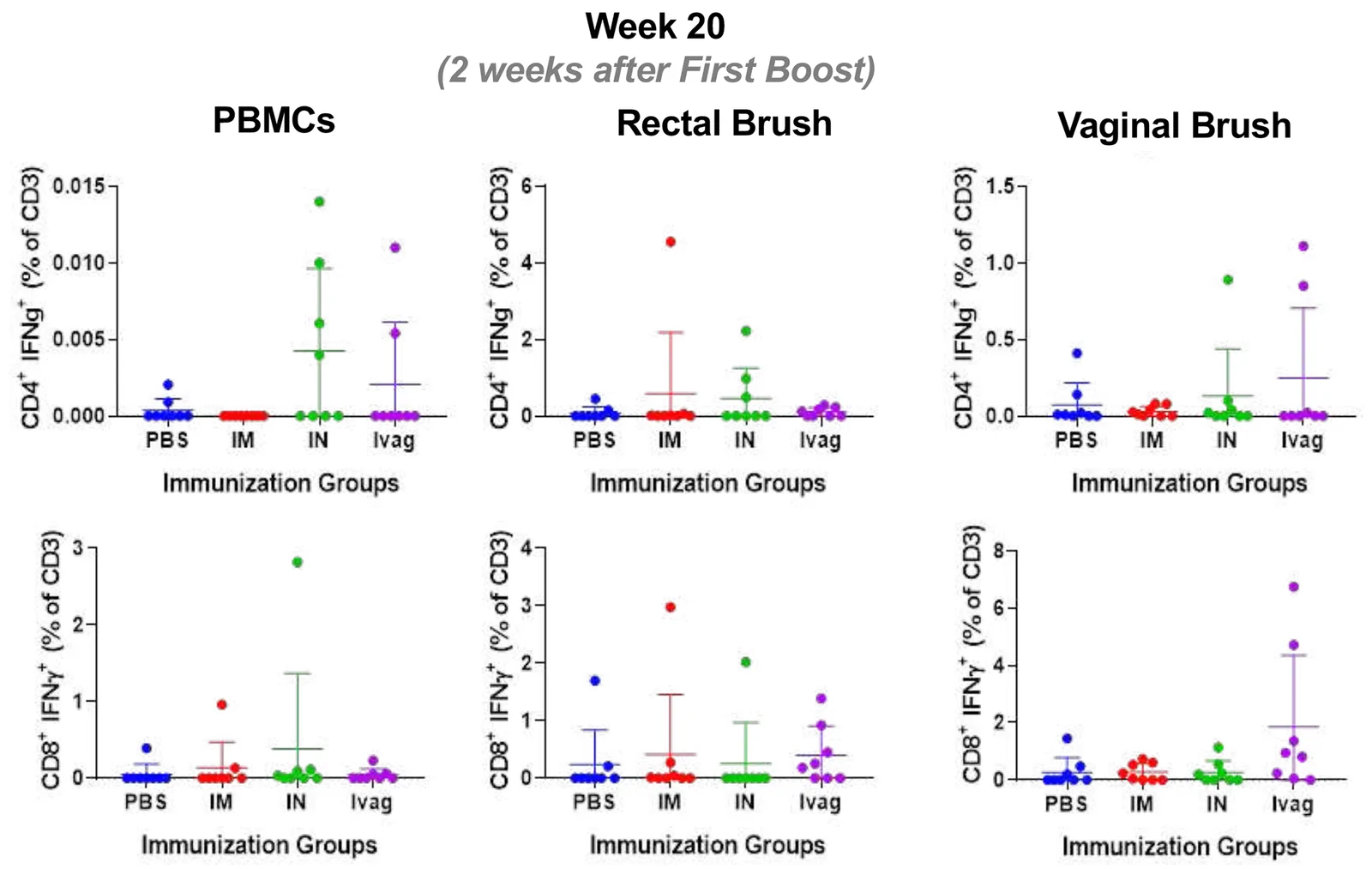
Flow Cytometric Analyses of Immune Cells in PBMCs and in Vaginal and Rectal Brush Samples 2 Weeks after Second Immunization. Samples were collected 2 weeks after the first vaccine boost and were analyzed without antigen stimulation by flow cytometry for the indicated markers.

Cellular responses were also assessed after two SC-Ad and SOSIP protein immunizations by collecting PBMCs and rectal and vaginal epithelial cells using our cytobrush method (88). Flow cytometry on these cells was performed without any additional antigen stimulation. This showed that a subset of animals had IFN-γ^+^ CD4 and CD8 cell responses after two immunizations (Fig. 6). There appeared to be some increased numbers of IFN-γ secreting CD8 cells in vaginal brush samples only in the IVAG-immunized animals. This was interesting, since these cells were not re-stimulated with antigen prior to flow cytometry, so they appeared to be activated and resident in the vaginal epithelium after two immunizations.

### Vaginal Immune Cells 82 weeks after Four SC-Ad and SOSIP Co-immunizations

**Figure 7.**
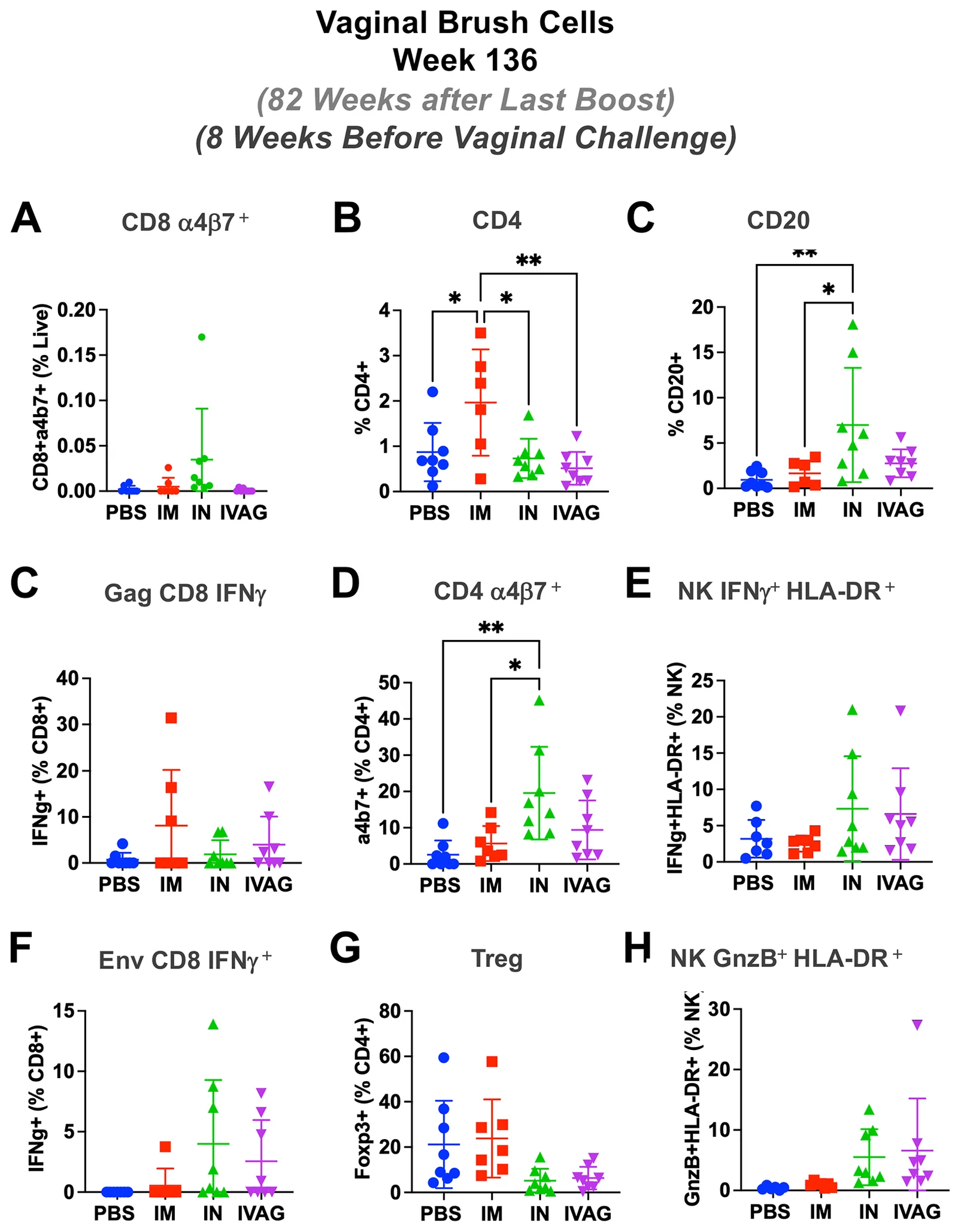
Flow Cytometric Analyses of Immune Cells in Vaginal Brush Samples 82 Weeks after Last Immunization. T cells were harvested from vaginal cytobrush samples 82 weeks after the last SC-Ad and SOSIP boost. These were analyzed by flow cytometry without any additional SIV or HIV antigen stimulation for the indicated cell markers. Each dot represents the mean value for each animal. * p < 0.05, ** p < 0.01 by one-way ANOVA.

Vaginal cytobrush samples were collected at week 136, 82 weeks after the last boost and 8 weeks before vaginal SHIV challenge. While this collection method yields only a small number of mucosal immune cells that limits assays to flow cytometry (88), it was chosen to limit damage to the vaginal mucosa before subsequent vaginal SHIV challenges. Flow cytometry was performed against week 136 vaginal brush cells without any antigen stimulation. These results showed the presence of a variety of CD4, CD8 in the vaginal mucosa more than a year and a half after last immunization (Fig. 7). IFN-γ+ Gag and env− specific and α4β7 integrin+ CD8 cells were observed in vaginal samples, but these did not reach significance due to the limited number of cells (Fig. 7A, C, and F). CD4 cells were significantly elevated in vaginal samples only in the IM SC-Ad plus IM SOSIP group hinting at potential higher density of viral targets (Fig. 7B). α4β7 integrin+ CD4 cells were significantly elevated in vaginal samples of the IN SC-Ad plus IM SOSIP group potentially enhancing susceptibility to virus acquisition in these cells (Fig. 7D). CD4 Treg cells were lower in vaginal samples in the IN and IVAG SC- groups but did not reach significance (Fig. 7G). NK cells were observed in some animals of the IN and IVAG groups but the difference from other groups was not significant (Fig. 7E and H). Vaginal CD20 B cells were significantly elevated only in the IN group (Fig. 7C).

### Repeated Vaginal Challenges with Clade C SHIV 90 Weeks After Last Immunization

Because of pandemic delays, the macaques were not challenged until 89 weeks after the last immunization. The macaques were repeatedly challenged with 10 weekly vaginal administrations of clade C SHIV.CH505.375H.dCT. The HIV-1 envelope in this challenge virus had 79.2% amino acid identity with the SOSIP protein vaccine and 75.4% identity with the envelope expressed by the SC-Ad vaccine (Supplemental Figure 1).

**Figure 8.**
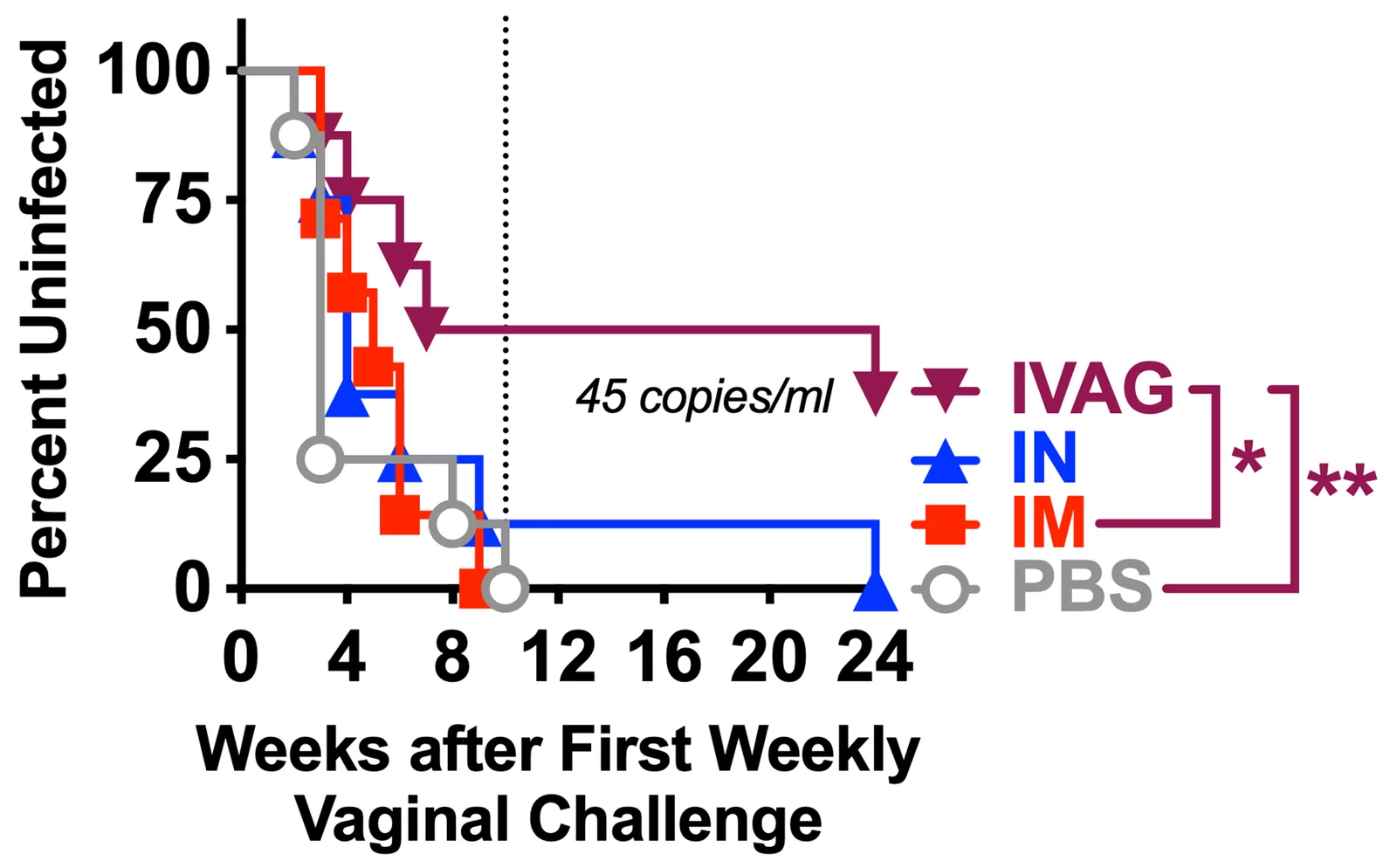
Protection against Repeated Vaginal SHIV Challenges. The indicated groups were challenged rectally with clade C SHIV CH505 on a weekly basis. Plasma samples were analyzed for SHIV viral RNA copies. Animals with RNA copies above the 45 lower limit of quantification (LLOQ) were considered infected. The number of challenges required to infect that animal was used as events for Kaplan-Meier survival analysis.

**Figure 9.**
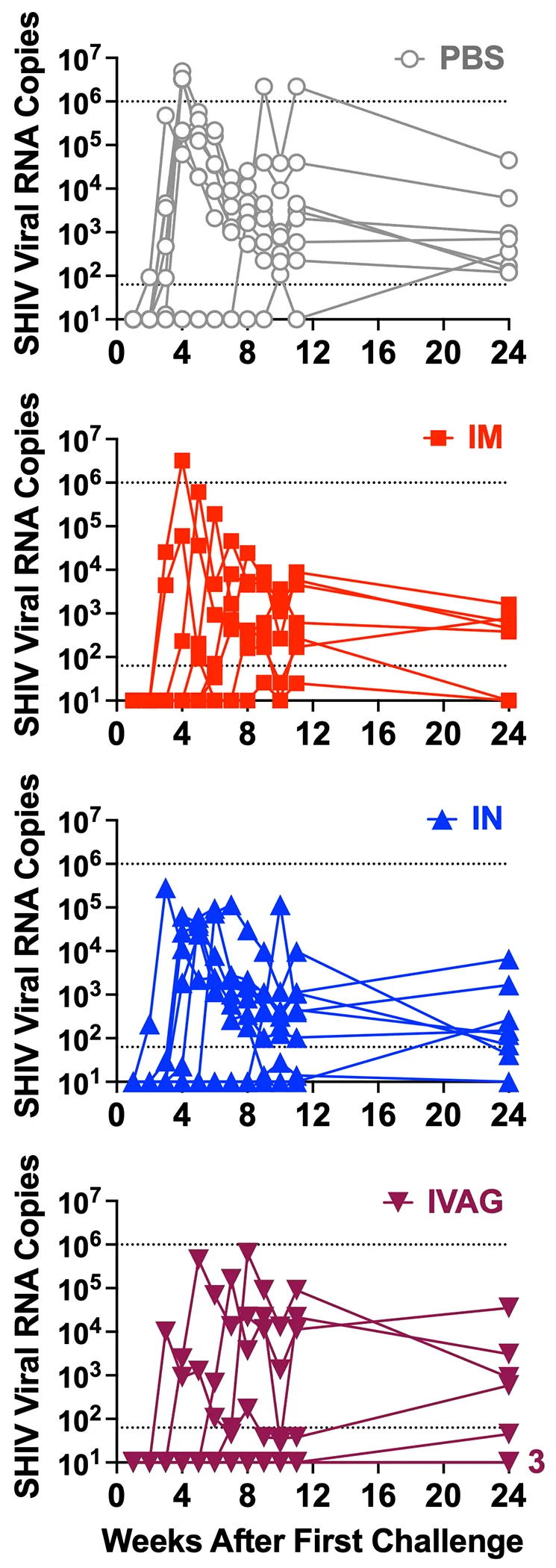
SHIV Acquisition and Viral Loads. Plasma CH505 viral RNA levels over the course of the challenge study.

**Figure 10.**
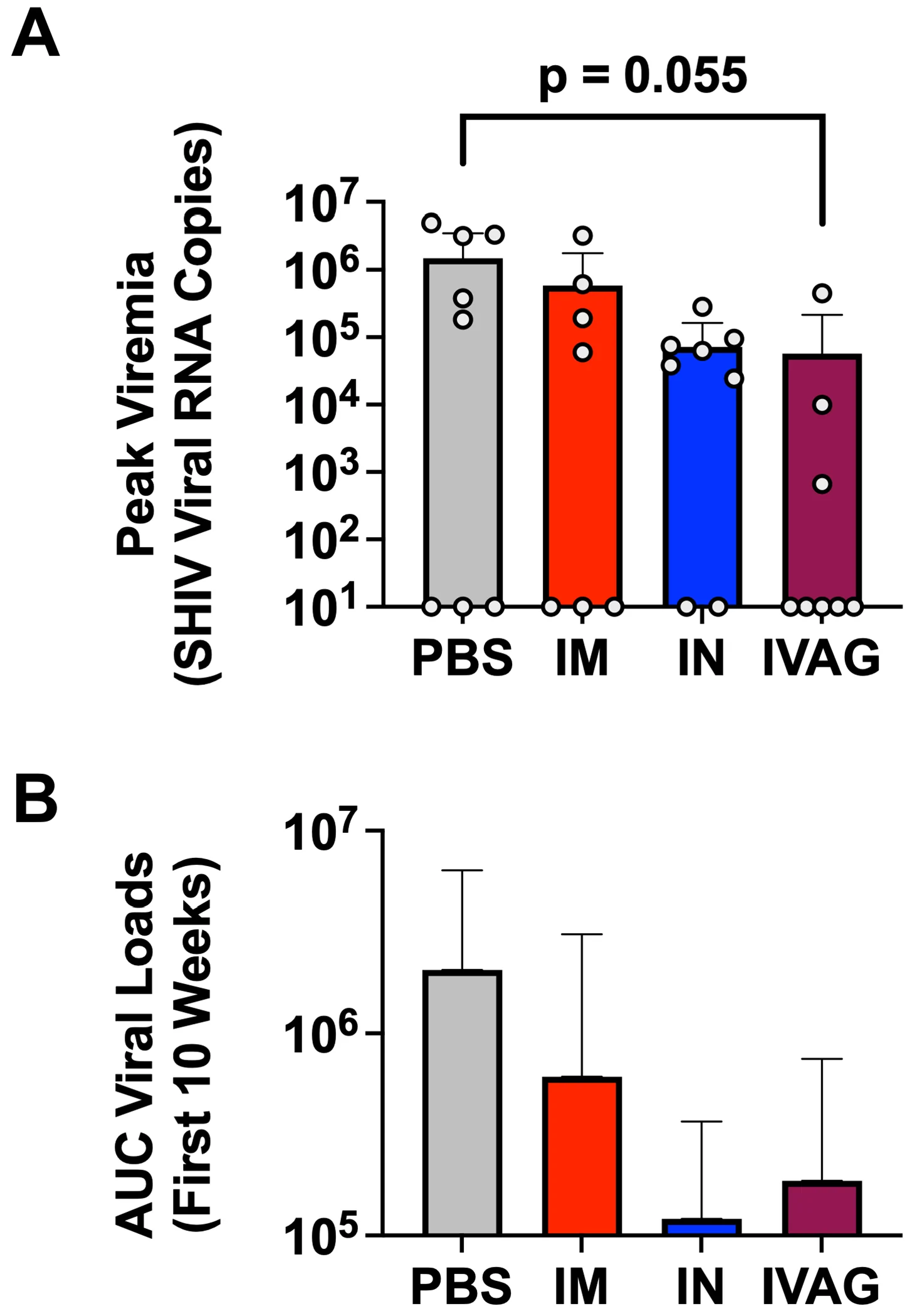
SHIV Peak and Cumulative Viremia during 10 Vaginal Challenges. **A)** Peak plasma CH505 viral RNA levels from Figure 9 were compared for each group by one way ANOVA. **B)** Area under the curve (AUC) for all of the animals from each group were calculated over the 10 week challenge period. AUC was not calculated through 24 weeks since samples were not measured between week 10 and 24 and might misrepresent viremia in the animals.

All animals in the PBS control and IM vaccine groups became infected within 10 challenges with a median of 4-5 exposures (Fig. 8). One IN group animal resisted the 10 infections, though the median number of exposures for this group was comparable to the previous two groups. In contrast, 50% of the IVAG SC-Ad-IM SOSIP group resisted all 10 vaginal challenges. A comparison of peak viral loads within these first 10 weeks showed no significant differences in IM and IN groups, but that the IVAG group achieved a p value of 0.055 by one way ANOVA (Fig. 9 and 10A). Area under the curve calculations for the first 10 weeks reflected higher viral loads in PBS than the vaccinated groups, with lowest levels in the 2 mucosal SC-Ad groups, but no group met statistical significance by this metric.

The animals were held until 24 weeks after challenge. At this time point, viral loads were measured showing that the one IN group animal that resisted the 10 challenges had SHIV RNA copies above 100 (Fig. 9). Similarly, one of the four SHIV resistant IVAG animal also had detectable SHIV RNA of 45 copies at week 24, suggesting that this one IVAG monkey may have had a late viral breakthrough. Three of eight IVAG SC-Ad plus IM SOSIP vaccinated animals still had undetectable SHIV viral RNA 24 weeks after first challenge or 114 weeks after last immunization.

## Discussion

The impetus for this work was borne from our past comparisons of how delivery routes of gene-based vaccines affected protection against mucosal pathogens. We previously showed that IN RD-Ad, SC-Ad and RC-Ad influenza vaccines were more protective against respiratory influenza challenges than IM Ads (89–92). Likewise, we showed that IN RD-Ad or SC-Ad provided better protection against (47). For HIV-1, we first showed that IVAG replication-defective helper-dependent Ad (HD-Ad) expressing only HIV-1 envelope was more protective than IM vaccine against rectal SHIV challenge (93).

Based on these observations, we hypothesized that protection against incoming pathogens will be most effective if vaccines are delivered at the site of virus entry. For example, the vast majority of COVID-19 vaccines are injected systemically when the infection targets the respiratory tract. Yet, when we compared RD- and SC-Ads against this virus, IN immunization was more effective against respiratory SARS-CoV-2 challenge (47).

For HIV-1, most infections occur at mucosal sites including the vaginal, rectal, penile, and oral mucosa. Targeting each of these sites would be challenging and impractical for clinical translation. We and others hypothesized that immunization of an accessible mucosal site might extend to other mucosal surfaces including those at-risk of HIV-1 infection (51, 94, 95). (23, 96, 97). In our hands, oral vaccines are often completely destroyed before reaching the immunization site, even if lyophilized and encapsulated (51, 94, 95). In contrast, IN immunization is simple and easily deployed and has the advantage of being a “needle-free” vaccine (29, 34).

When we tested IN vs IM immunization with SC-Ad followed by protein boost against clade B SHIV, IN immunization generated weak responses in the systemic compartment, but provided subtle benefits in the quality of mucosal immune responses (50). Despite this, these vaccines failed to protect against repeated rectal clade B SHIV challenges. The only suggestion of success were subtle reductions in SHIV viral loads in the digestive tract in IN SC-Ad immunized animals.

Encouraged by these observations, this study aimed to compare an improved immunization protocol via IM and IN immunizations against clade C SHIV. However, we also included a “positive control” vaccine route wherein the gene-based vaccine was delivered in the vagina, the very site of later SHIV challenges. We pivoted to testing against clade C HIV env in this study to better match prevailing global infections and we updated the protein vaccine with a trimeric SOSIP immunogen.

Because SOSIPs had at that point only been tested by IM routes, this protein vaccine was delivered only by the intramuscular route. The only vaccines whose routes were varied were the gene-based replicating SC-Ad vaccines. Therefore, if there were no effects of SC-Ad route on immune responses or protection, one could infer that these gene-based vaccines were not contributing to these effects or protection against SHIV. If all animals had similar immune responses and protection against SHIV challenge one might conclude that these effects were mediated by only the protein vaccine or by the protein vaccine being assisted by the SC-Ads in a route independent fashion.

The data from this study suggest that route and the gene-based vaccines both play important roles in the quality and magnitude of immune responses at specific locations as well as in the ability to repel incoming vaginal clade C SHIV. IM and IVAG SC-Ad immunized animals induced stronger immune systemic and mucosal responses than animals immunized by the IN route. This is not surprising since IM and IVAG both had the advantage of being injected into tissues, whereas the IN vaccines were administered as deposited liquids. Much of this liquid IN vaccine likely does not infect cells and runs down the throat to GI tract where it likely gets destroyed.

While IM and IVAG both generated similarly strong antibody and cellular immune responses in their systemic compartment (plasma and PBMCs), all IM immunized animals became infected by clade C SHIV whereas 50% of the IVAG group resisted 10 vaginal SHIV challenges pointing at qualitative differences in the correlates of protection. Indeed, neutralizing antibodies were highest in the plasma of the IM group, yet these animals were less protected against vaginal SHIV than the other vaccinated groups. Also, IN and IVAG animals that resisted the 10 SHIV infections did not have higher plasma neutralizing antibodies than those that succumbed (data not shown).

Systemic T cell responses showed a large degree of variability within groups, though a majority of animals (7/8) in the IVAG group retained IFN-γ responses at week 136 without any SIV or HIV antigen restimulation when compared to the other groups. Even though the differences between the groups were not statistically different, this was suggestive of additional correlates of protection. However, the IN or IVAG animals that resisted the 10 infections did not have notably higher ELISPOT responses in PBMCs than those that were infected (data not shown).

Similarly, vaginal cell mediated responses suggested that the IVAG group as the only one with CD8 response against both antigens, though this difference again was not absolute (Fig. 7C and F). The latter responses have to be taken into account with the caveat of very limited cells available to run these assays. Protective responses also could have been counterbalanced by the recruitment or presence of higher number of CD4+ target cells in the IM group (Fig. 7B) and α4β7+CD4+ cells in the IN group (Fig. 7D)(98).

Most impressive for this current study was the observation of partial protection after 1.5 year beyond the last immunization. While the COVID pandemic markedly altered our experimental schedule, this allowed for the demonstration of durable immunity subsequent of our immunization strategy. It is unclear whether earlier challenges might have been met with a better protection outcome.

Of all of the variables entering this study, the primary correlate of protection was the site of SC-Ad vaccination. The SOSIP protein vaccine was uniformly administered IM across all immunized groups. Since protection tracked with the site of SC-Ad vaccination, it appears that the gene-based vaccine mediated important roles in providing barrier protection against incoming clade C SHIV. Whether the protein vaccine contributed to this protection is not clear from this study. However, in our previous study with clade B SC-Ad and gp140 protein boost, there were remarkable increases in immune correlates after protein boost, so we infer that the SOSIP vaccine was an important contributor to protection in this study.

Whether SOSIP alone or in combination with SC-Ad might also be more potent by the IVAG route remains to be determined. Previous immunizations protocols using clade A SOSIP adjuvanted with other adjuvants e.g. 3M-052 provided excellent and durable antibody responses (99)), suggesting that the combination with Adjuplex might have been suboptimal, at least in terms of immunogenicity in our experiment.

Nevertheless, these data suggest that there are indeed merits to building barriers of protection at mucosal sites of pathogen entry, and moreover the ability to induce long lasting protective responses. This data for vaginal uptake of clade C SHIV is consistent with our data with IN SC-Ad immunization leading to better protection against respiratory SARS-CoV-2 infection. The challenge for making use of this observation for HIV is that we cannot practically deploy a vaccine that must be injected in the vaginal, rectal, or penile tract. Given our observations of the “distance effect” and rampant destruction of oral vaccines, we think IN or oral vaccines are unlikely to fill this gap. Clearly, alternative administration modalities need to be devised to obtain the same type of mucosal protection that was observed in this study. For example, subcutaneous or intramuscular immunizations that target antigens to the same lymph nodes that drain the female and male reproductive tracts, or to devise a less invasive mucosal delivery methods that might be feasible to deploy in humans.

## Supplementary Material

1
