# Supplementary material for: Comparison of Systemic and Mucosal Synchronous Immunization with Replicating Single-cycle Adenoviruses and SOSIP Protein HIV-1 Vaccines
Source: Res Sq. 2026 Aug 26:rs.3.rs-10702892. Preprint. [Version 1] doi: 10.21203/rs.3.rs-10702892/v1 (PMC13532745; doi:10.21203/rs.3.rs-10702892/v1)
Supplement: Supplementary file 1 [file NIHPPrs10702892v1-supplement-1.pdf]

Supplemental Figure 1. Envelope Amino Acid Sequence Alignments of SC-Ad and SOSIP Immunogens and CH505 Challenge Virus. ClustalW alignment of the sequences was performed by MacVector.

Supplemental Figure 1

```
SHIV C.CH505 gp160   NGMWVTVYYGVPVWKEAKTTLFCASDAKAYEKEVHNVWATHACVPTDPNPQEMVLKNVTE
SC-Ad Clade C gp160  GNLWVTVYYGVPVWKEAKTTLFCASDAKAYEKEVHNVWATHACVPTDPNPQEIVMGNVTE
Clade C SOSIP        GNMWVTVYYGVPVWTDAKTTLFCASDTKAYDREVHNVWATHACVPTDPNPQEIVLENVTE

SHIV C.CH505 gp160   NFNMWKNDMVDQMHEDVISLWDQSLKPCVKLTPLCVTLNCTNATASNS------------
SC-Ad Clade C gp160  NFNMWRNDMVEQMHEDIISLWDESLKPCVKLTPLCVTLHCTNATVTNANATSNGTATYNN
Clade C SOSIP        NFNMWKNDMVDQMHEDIISLWDQSLKPCVKLTPLCVTLHCTNATFKNN---------VTN

SHIV C.CH505 gp160   SIIEGMKNCSFNITTELRDKREKKNALFYKLDIVQLD-----GNSSQYRLINCNTSVITQ
SC-Ad Clade C gp160  TGNGEMKNCSFSTTTEIRDKKQQAYALFYKPDIVLLN-----KTSSEYILINCNSSTITQ
Clade C SOSIP        DMNKEIRNCSFNTTTEIRDKKQQGYALFYRPDIVLLKENRNNSNNSEYILINCNASTITQ

SHIV C.CH505 gp160   ACPKVSFDPIPIHYCAPAGYAILKCNNKTFTGTGPCNNVSTVQCTHGIKPVVSTQLLLNG
SC-Ad Clade C gp160  ACPKVSFDPIPIHYCAPAGYAILRCNNKTFNGTGPCHNVSTVQCTHGIKPVVSTQLLLNG
Clade C SOSIP        ACPKVNFDPIPIHYCAPAGYAILKCNNKTFSGKGPCNNVSTVQCTHGIKPVVSTQLLLNG

SHIV C.CH505 gp160   SLAEGEIIIRSENITNNVKTIIVHLNESVKIECTRPNNKTRTSIRIGPGQAFYATGQVIG
SC-Ad Clade C gp160  SLAEGEIIIRSENITNNAKTIIVQLNESVAINCTRPNNNTRKSIRIGPGQTFYATGDIIG
Clade C SOSIP        SLAEKEIIIRSENLTDNVKTIIVHLNKSVEIVCTRPNNNTRKSMRIGPGQTFYATGDIIG

SHIV C.CH505 gp160   DIREAYCNINESKWNETLQRVSKKLKEYFPH-KNITFQPSSGGDLEITTHSFNCGGEFFY
SC-Ad Clade C gp160  DIRQAHCNISRQSWNTTVERIKKKLQEHFPN-ATIEFKPHAGGDLEITTHSFNCRGEFFY
Clade C SOSIP        DIRQAYCNISGSKWNETLKRVKEKLQENYNNNKTIKFAPSSGGDLEITTHSFNCRGEFFY

SHIV C.CH505 gp160   CNTSSLFNRTYMANSTDMANSTETNSTRTITIHCRIKQIINMWQEVGRAMYAPPIAGNIT
SC-Ad Clade C gp160  CNTTKLFNTNNSD----------SDNTTTITLPCRIKQIANLWQGVGRAMYAPPIAGNIT
Clade C SOSIP        CNTTRLFNNN-------------ATEDETITLPCRIKQIINMWQGVGRAMYAPPIAGNIT

SHIV C.CH505 gp160   CISNITGLLLTRDGGK----NNTETFRPGGGNMKDNWRSELYKYKVVEVKPLGVAPTNAR
SC-Ad Clade C gp160  CNSNITGLLLTRDGGRGINESETETFRPGGGDMRDNWRSELYKYKVVEIKPLGIAPTASK
Clade C SOSIP        CKSNITGLLLVRDGGE--DNKTEEIFRPGGGNMKDNWRSELYKYKVIELKPLGIAPTGAK

SHIV C.CH505 gp160   RRVVEREKR-AVGMGAVFLGFLGAAGSTMGAASITLTVQARQLLSGIVQQQSNLLKAIEA
SC-Ad Clade C gp160  RRVVEREKRAVVGIGAVFLGFLGAAGSTMGAASLTLTVQARQLLSGIVQQQSNLLRAIEA
Clade C SOSIP        R---------AVGIGAVFLGFLGAAGSTMGAASLTLTVQARQLLSSIVQQQSNLLRA-EA

SHIV C.CH505 gp160   QQHMLKLTVWGIKQLQARVLALERYLKDQQLLGMWGCSGKLICTTNVYWNSSWSNKTYGD
SC-Ad Clade C gp160  QQHMLQLTVWGIKQLQARVLAIERYLKDQQLLGLWGCSGKIICTTAVPWNASWSNKSKED
Clade C SOSIP        QQHMLQLTVWGIKQLQTRVLAIERYLKDQQLLGIWGCSGKLICTN-VPWNSSWSNKSQTD

SHIV C.CH505 gp160   IWDNMTWMQWEREISNYTEIIYELLEESQNQQEKNEQDLLALDRWNSLWNWFNITNWLWY
SC-Ad Clade C gp160  IWNNMTWMQWDIEISNYTNSIYQLLEESQTQQEQNEKDLLALDKWQNLWNWFDITNWLWY
Clade C SOSIP        IWNNMTWMEWDREISNYTDTIYRLLEDSQTQQEKNEKDLLALD-----------------

SHIV C.CH505 gp160   IKIFIMIVGGLIGLRIIFAVLSLVNRVRQGYSPLSLQTLLPTPRGPARPEGIEEEGGEQG
SC-Ad Clade C gp160  IKIFIMIVGGLIGLRIIFAVLSIINRVRQGYSPLSFQTLTPTPRGPDRHEGIEEEGGEQG
Clade C SOSIP        ------------------------------------------------------------

SHIV C.CH505 gp160   RDRSIRLLTGLSELIWDDLRNLCLFSYHHLRDLILIAARIVQLLGR-------RGWEALK
SC-Ad Clade C gp160  RDRSVRLVNGFLSLAWDDLRNLCLFSYRHLRDFILVIARVVELLGHSSLRGLQRGWEALK
Clade C SOSIP        ------------------------------------------------------------

SHIV C.CH505 gp160   YLWNILQYWIQELKNSAISLFDTIAIAVAYLQYGWSYFHEAVQAGWRSATETLAGAWGDL
SC-Ad Clade C gp160  YLGSLVQYWGLELKKSALSLLDTIAIAVAEGTD-------------------------RI
Clade C SOSIP        ------------------------------------------------------------

SHIV C.CH505 gp160   WETLRRGGRWILAIPRRIRQGLELTLL*
SC-Ad Clade C gp160  IEFLQRVCRAIRNLPRRIRQGFEAALL
Clade C SOSIP
```

	SHIV	SC-Ad	SOSIP
SHIV	-	75.4%	79.2%*
SC-Ad		-	81.4%*
SOSIP			-

* alignment to only SOSIP region
